# Reduced 30-day mortality in men after elective coronary artery bypass surgery with minimized extracorporeal circulation-a propensity score analysis

**DOI:** 10.1186/1471-2261-12-17

**Published:** 2012-03-16

**Authors:** Michael Ried, Reinhard Kobuch, Leopold Rupprecht, Andreas Keyser, Michael Hilker, Christof Schmid, Claudius Diez

**Affiliations:** 1 Department of Cardiothoracic Surgery, University Medical Center Regensburg, Franz-Josef-Strauß-Allee 11, Regensburg 93053, Germany

**Keywords:** Outcome, Men, MECC, CABG, Mortality, Propensity score analysis

## Abstract

**Background:**

Impact of minimized extracorporeal circulation (MECC) for coronary surgery on mortality remains controversial and gender significantly influence outcome.

**Methods:**

We analyzed 3,139 male patients undergoing elective coronary surgery between 01/2004 and 05/2009. Using propensity score matching after binary logistic regression, 1,005 patients (from 1,119 patients) undergoing surgery with MECC could be matched with 1,005 patients (from 2,020 patients) undergoing surgery with conventional extracorporeal circulation (CECC). Primary outcome was 30-day mortality.

**Results:**

Unadjusted 30-day mortality was 2.7% in patients with CECC and 0.8% in those with MECC (mean difference -1.9%; p < 0.001). The adjusted mean difference (average treatment effect of the treated) after matching was -1.5% (95% confidence interval (CI) -2.6 to -0.4; p = 0.006). Postoperative hospital stay was shorter in patients operated with minimized systems (adjusted mean difference -0.8 days; 95% CI -1.46 to -0.09; p = 0.03) and incidence of postoperative neurocognitive dysfunction was also lower (adjusted mean difference -1.3%; 95% CI -2.2 to -0.4; p = 0.001). Chest tube drainage (adjusted mean difference +22 mL; 95% CI -47 to 91; p = 0.5) and risk for acute kidney injury, kidney injury and failure according to RIFLE criteria (adjusted mean difference -1.0%; 95% CI -2.5 to 0.6; p = 0.24) proved to be insignificant between both groups. Apart from reduced 30-day mortality, however, average treatment effects for intensive care unit stay, postoperative hospital stay, chest tube drainage and kidney injury did not significantly differ.

**Conclusion:**

Using propensity score analysis, we observed an association between MECC and reduced 30-day mortality in men, but our results call for further analysis.

## Background

Coronary artery bypass grafting (CABG) with conventional extracorporeal circulation (CECC) remains the treatment of choice, in particular, for multivessel disease [1,2]. Common side effects of CECC include systemic inflammatory response and dysfunctional coagulation pathways leading to potential end-organ failure. Although the concept of minimized extracorporeal circulation (MECC) was introduced more than ten years ago, it has not gained widespread use. Whereas some studies could demonstrate improved survival, reduced systemic inflammation and reduced transfusion requirement after CABG with MECC [3,4], other reports failed to confirm these results [5]. Limitations of many recent studies arose from observational study design and limited number of patients. Both facts lead to biased (i.e. selection bias) results and lack of power, which in turn made definitive conclusions for MECC use difficult, in particular for determining postoperative mortality [6]. Our institution has been using MECC for more than ten years and we could show that MECC is a safe alternative extracorporeal circulation (ECC) system and even high-risk patients can undergo CABG with MECC [7].

Gender significantly impacts outcome after CABG and women were shown to have increased mortality and morbidity even after risk adjustment [8]. However, the reasons for gender-related differences in outcome after CABG surgery still remain controversial and vastly unknown, but agreement exists on strong gender-related bias in observational trials [9]. Most studies that examined gender differences in CABG surgery compared conventional ECC with off-pump coronary artery bypass grafting or analyzed it in patients undergoing CABG with conventional ECC.

The objective of this study was to evaluate the impact of minimized ECC and CECC use on mortality in males undergoing elective CABG. A propensity score based approach was used to estimate treatment effects of MECC in men. We excluded women because it has been previously shown that matching efficiency was very low (26%) if propensity scores are used which contain gender as a covariate [10]. This fact is reflective of the vastly dissimilar preoperative profiles of women and men and thus leads to little overlap of scores, which limits the number of matching pairs.

## Methods

### Patients and study design

This is an observational retrospective study with 3,139 male patients, who underwent elective coronary artery bypass grafting with conventional or minimized extracorporeal circulation between January 2004 and May 2009 at the University Medical Center Regensburg. The study was approved by the university's ethics board, the individual patient consent was waived because of the study's retrospective design and data collection from routine care. Exclusion criteria were as follows: emergent procedures, off-pump revascularization, redo surgery and preoperative renal replacement therapy. Patients with severe aortic regurgitation and a body mass index BMI ≥ 35 kg × m^-2 ^were contraindications for MECC use.

To avoid strong gender-related bias, we excluded female patients from this study.

### Data collection, variable definitions and study endpoints

Data were collected in and extracted from the institution's database and from medical records. Variables were defined according the European System for Cardiac Operative Risk Evaluation (EuroSCORE) and perioperative mortality was calculated with the logistic version of this risk model [11]. Serum creatinine was measured in mg/dL at the day of admission and glomerular filtration rate (in mL × min^-1 ^× 1.73 m^-2^) was estimated with the MDRD-equation (eGFR = 186.3 × SCr^-1.154 ^× age^-0.203^) [12].

Primary endpoint was 30-day mortality. Secondary outcome variables were intensive care unit stay, postoperative stay, acute kidney injury (AKI) and severe neurocognitive dysfunction (prolonged ischemic neurologic deficit (PRIND), stroke). The RIFLE criteria [13] were used to estimate acute kidney failure within the first 72 postoperative hours. The highest serum creatinine value measured in this time was used to calculate relative changes to baseline creatinine before operation and to assign a patient into the appropriate group (risk, injury and failure). The term "reperfusion time" refers to the span between end of myocardial ischemia and end of cardiopulmonary bypass irrespective of CECC or MECC use.

### Surgical procedure

After full median sternotomy, patients were connected to the MECC system (MAQUET-System, Hechingen, Germany), a fully closed circuit without blood-air contact. Heparin (125 IE/kg) was administered following preparation of left internal mammary artery (LIMA) to target an activated clotting time (ACT) between 250 to 300 seconds. Calafiore's blood cardioplegia was used for cardiac arrest in all MECC patients. Further technical details have been previously reported [14].

For conventional ECC heparin (350 IE/kg) was administered after LIMA harvest and an ACT of ≥ 450 seconds was targeted. Cardiac arrest was achieved with Bretschneider's solution in 96% of patients undergoing CABG with CECC.

The ascending aorta and the right atrium (with a two-stage cannula) were cannulated in all patients. All operations were performed with mild hypothermia (34°C). After surgery all patients were transferred to the intensive care unit and received standard hemodynamic monitoring.

All operations were performed by six senior cardiac surgeons, who were experienced with both MECC and CECC. The proportion of MECC procedures did not significantly differ between surgeons and all also operated a similar proportion of patients with CECC. Impaired quality of coronary anatomy, diffuse vessel pathology or more distal stenoses did not exclude MECC use. Only 9.4% (n = 190) of CECC patients were training procedures for residents and resulted in a slightly lower use of LIMA. The final decision whether to use MECC or not was left to the surgeon.

### Statistical analysis

Statistical analysis was done with Stata 10.1 SE (StataCorp., College Station, USA). Stata's module *psmatch2 *[15] was used for propensity score matching and covariate imbalance testing. Continuous variables were first tested for normality with the Shapiro-Wilk test and with Quantile-Quantile-plots. If normally distributed, they are presented as means ± standard deviations, otherwise as median with interquartile range (25^th ^and 75^th ^percentile). Student's *t*-test was used for comparison of two continuous, normally distributed variables, whereas Wilcoxon's ranksum test was used for non-normally distributed variables. Categorical data were shown as frequency distributions and analyzed with Fisher's exact test (in a 2 × 2 table) or with the Chi-square test.

Because patients in this study were not randomly assigned to CABG with MECC, we matched patients based on their propensity (conditional probability) to undergo CABG with MECC. The propensity score (PS) is a subject's probability of receiving a specific treatment conditional on the observed covariates [16]. The propensity score was calculated by binary logistic regression including all variables marked with an asterisk in Table 1. Nearest neighbor matching with a caliper ε = 0.2 × σ_P _(σ_P _denotes standard deviation of the estimated PS) was used to match 1,005 patients in the MECC group to 1,005 patients from the ECC group (matching efficiency 89.8%; 1,005/1,119 of MECC patients; 1,005 pairs). We used non-replacement that is, once a treated case is matched to one non-treated case, both cases were removed from the pool. We defined logit = log ((1-PS)/PS) as propensity score and used it for matching. The logit of PS is called linear predictor of the PS.

**Table 1:** Unadjusted pre- and operative baseline data

Variable	CECC (n = 2,020)	MECC (n = 1,119)	p-value
Age [years]	65.5 ± 8.7	66.1 ± 8.8	0.05*
Age group [n;%]			
< 59	469 (23)	250 (22)	0.06
60-69	821 (41)	435 (39)	
70-79	688 (34)	393 (35)	
> 80	42 (2)	41 (4)	
Logistic EuroSCORE [%, 95% CI)^A^	3.2 (3.0 to 3.4)	3.2 (3.0 to 3.4)	0.16
No of distal anastomoses [n]	3 (3; 4) Range (1-8)	3 (3; 4) Range (1-6)	< 0.001*
Use of LIMA [%]	88.3	93.6	< 0.001*
Bypass time [min]	95 ± 31	84 ± 26	< 0.001*
Aortic cross clamp time [min]	57 ± 19	51 ± 17	< 0.001*
Reperfusion time [min]	34 ± 12	28 ± 12	< 0.001*
Ejection fraction [%]	62 (49; 70)	63 (50; 70)	0.12
Body mass index [kg × m^-2^]	28.4 ± 3.8	28.2 ± 3.5	0.65*
Body surface area [m^2^]	1.97 ± 0.16	1.95 ± 0.14	< 0.001*
Hemoglobin [mg × dL^-1^]	13.0 ± 1.5	13.1 ± 1.5	0.02*
Leukocytes [n × 10^-9 ^L]	7.9 (6.7; 9.3)	7.7 (6.6; 9.2)	0.16
Thrombocytes [n × 10^-9 ^L]	251 (210; 290)	248 (206; 292)	0.06
Arterial hypertension [%]	74.4	79.9	< 0.001*
COPD [%]	8.6	9.0	0.69
Atrial fibrillation [%]	3.8	3.3	0.48
Peripheral vascular disease [%]	6.9	6.0	0.32
Insulin-dependent diabetes [%]	27.5	28.8	0.45
Serum creatinine [mg × dL^-1^]	1 (0.9; 1.2)	1 (0.9; 1.1)	0.15
Estimated GFR^B ^< 60 mL × min^-1 ^× 1,73 m^-2 ^[%]	16.4	18.4	0.14

^A^*CI *confidence interval

^B^*GFR *glomerular filtration rate

**Figure 1:**
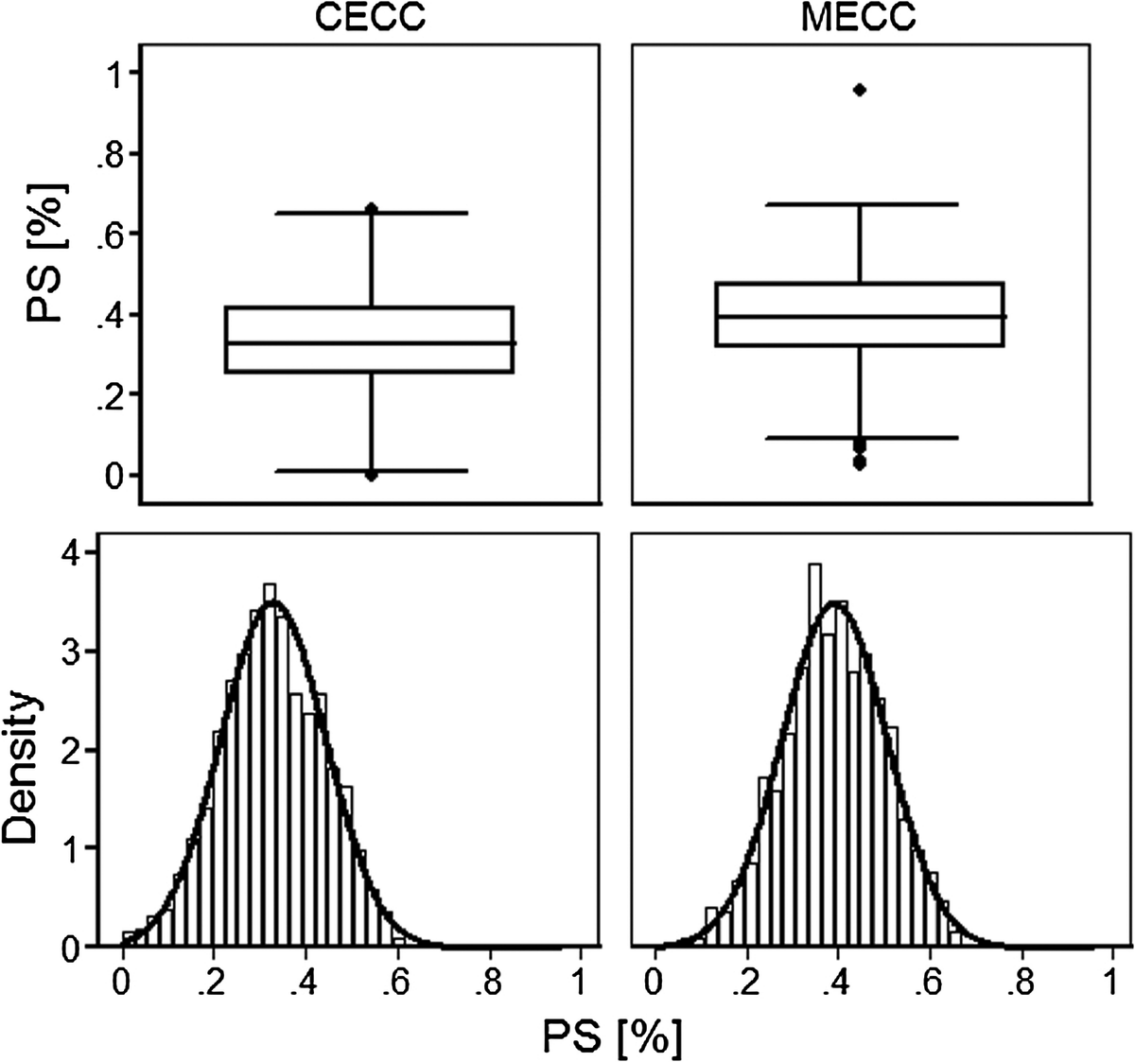
**Distribution of estimated native propensity score (not the logit of PS) stratified to type of extracorporeal circulation**. There is a sufficient overlap of propensity scores between both groups. The lower panels show the density distributions of PS and the superimposed normal curves. CECC-conventional extracorporeal circulation; MECC-minimized extracorporeal circulation; PS-propensity score.

Before matching the mean PS for MECC use in men operated with conventional ECC (n = 2,020) was 0.3281 ± 0.1142 and in those receiving CABG with MECC 0.3914 ± 0.1145 with an associated standardized difference of 55% (95% CI 48 to 63; *t *test p-value < 0.001). After matching, the mean PS for MECC use in the matched patients not receiving MECC was 0.3827 ± 0.1049 and in those receiving MECC was 0.3875 ± 0.1108, which yielded a standardized difference of 4.2% (95% CI -4.3 to 13.1; p > 0.05 for a two-tailed test). Figure 1 displays the distributions of estimated propensity scores stratified to treatment (MECC) or control (CECC) group.

Covariate balance was tested using the standardized difference before and after matching together with the achieved percentage reduction in absolute bias. The standardized difference is the difference of the sample means in the treated and non-treated sub-samples as a percentage of the square root of the average of the sample variances in the treated and non-treated groups.

The average treatment effect for the treated (ATT) answers the question: Is MECC beneficial for those individuals, who were (not randomly) assigned to treatment, i.e. MECC and was estimated with Wilcoxon's signed rank test for continuous variables or with McNemar's test for risk differences for binary outcomes to account for the paired data structure after matching. We also calculated the average treatment effect (ATE) that answers the question if MECC is beneficial for those randomly drawn from the overall population. Bootstrapping (400 replications) was used to estimate 95% CIs for ATEs. The key notion is ATT ≠ ATE and they are linked via ATE = N_1_/N × ATT + N_0_/N × ATU (N_0 _denotes the number of non-participants, N_1 _denotes the number of participants and ATU the average treatment effect for the non-participants).

Hidden bias was estimated according to Rosenbaum's sensitivity analysis. We used Stata's module *rbounds *[17] for this test.

A p-value < 0.05 was considered significant.

## Results

### Patient characteristics

Unadjusted baseline data were summarized in Table 1. Several pre- and perioperative covariates were significantly different between both groups and may reflect the non-randomized design of this study. Patients with MECC were slightly older, had a slightly lower median number of distal anastomoses, a higher frequency of LIMA use, shorter mean aortic cross-clamping and reperfusion time, lower mean body surface areas and a higher prevalence of arterial hypertension.

After matching, treated patients were similar with regards to all baseline covariates used for PS estimation (Table 2). Matching reduced covariate imbalance and improved covariate balance across treatment groups. The median absolute bias before matching was 17.6 and after matching 1.43.

**Table 2:** Covariate balance testing between unmatched and matched sample

Variable	Standardized difference [%]		% reduction |difference|	p-value	
	Unmatched	Matched		Unmatched	Matched
Age	8.1	0.1	98.3	0.03	0.97
No of grafts	-21.5	3.9	82.1	< 0.001	0.37
LIMA use	17.6	0.7	96.0	< 0.001	0.86
ECC-time^A^	-38.3	1.4	96.5	< 0.001	0.72
Cross clamp time	-26.9	3.3	87.8	< 0.001	0.42
Reperfusion time	-39.4	-1.5	96.3	< 0.001	0.68
Body mass index	1.5	1.0	32.6	0.65	0.61
Body surface area	-16.6	4.0	76.0	< 0.001	0.36
Hypertension	15.2	-1.4	90.6	< 0.001	0.74

^A^*ECC *extracorporeal circulation

### Outcome

The unadjusted 30-day mortality was 2.7% (55/2,020) in the conventional ECC group and 0.8% (9/1,119) in the MECC group (mean difference -1.9%; p < 0.001). The adjusted mean difference in 30-day mortality after matching confirms a survival benefit in patients, who were primarily assigned to MECC (-1.5%; 95% CI -2.6 to -0.4; p = 0.006). If randomly drawn from the overall population, CABG with MECC even exerts a survival benefit (ATE) of -1.9% (95% CI -2.9 to -1.0) because the mean ATU was 2.2% and thus enforced the ATE.

Secondary outcome variables were summarized in Table 3. Whereas intensive care unit stay was insignificant after adjustment for covariates, postoperative hospital stay was significantly lower in patients primarily assigned to MECC (ATT). The observed effect, however, was only marginal because the upper limit of the 95% confidence interval almost touches zero or includes zero (for the ATE). Severe neurocognitive dysfunction was less likely to occur in the MECC group with an adjusted mean difference of -1.3% (95% CI -2.2 to -0.4). Mean chest tube drainage was insignificant between treatment (MECC; 828 mL) and control group (CECC; 806 mL) after matching (adjusted mean difference +22 mL; 95% CI -47 to 91 mL; p = 0.50). The ATE for chest tube drainage was +36 mL (95% CI -22 to 97 mL; p > 0.05 for a two-tailed test).

**Table 3:** Estimated average treatment effects on several outcome variables

Comparison	Outcome measures			
ATT^A^	30-day mortality [%]	ICU stay [days]	Postoperative hospital stay [days]	Postoperative neuro-cognitive dysfunction [%]
CECC versus MECC				
CECC (n = 2,020)	2.7	2.6	12.9	1.1
MECC (n = 1,119)	0.8	2.2	12.0	0.2
Unadjusted mean difference	-1.9	-0.4	-0.9	-0.9
p-value	< 0.001	< 0.001	< 0.001	0.003
Adjusted mean difference after matching of 1,005 pairs (95% confidence interval)	-1.5 (-2.6 to -0.4)	-0.38 (-0.80 to 0.00)	-0.81 (-1.46 to -0.09)	-1.3 (-2.2 to -0.4)
p-value	0.006	0.050	0.03	0.001
ATE^B^				
Adjusted mean difference with 95% bias corrected confidence interval	-1.9 (-2.9 to -1.0)^C^	-0.36 (-0.72 to 0.01)^D^	-0.74 (-1.31 to 0.1)^D^	-1.0 (-1.70 to -0.4)^C^

^A ^- *ATT *Average treatment effect for the Treated

^B ^- *ATE *Average treatment effect

^C ^- The 95% confidence interval does not include a zero, or p < 0.05 for a two-tailed test

^D ^- The 95% confidence interval does include a zero, or p > 0.05 for a two-tailed test

The unadjusted requirement of PRBC transfusion did not differ between both groups (Table 4).

**Table 4:** Unadjustedperi- and postoperative data

Variable	CECC (n = 2,020)	MECC (n = 1,119)	p-value
Postoperative dialysis during entire hospital stay [%]	2.4	1.2	0.015
Drain loss [mL]	600 (490; 900)	650 (480; 950)	0.013
Frequency of PRBC^A ^transfusion [%]	44.7	41.7	0.10
No of transfused PRBC [n]	2 (1; 4)	2 (1; 3)	0.001
Frequency of FFP^B ^transfusion [%]	19.3	21.8	0.09
No of transfused FFP [n]	4 (3; 7)	5 (3; 8)	0.11
Ventilation time [min]	12 (8; 16)	11 (8; 15)	0.11

^A^*PRBC *packed red blood cells

^B^*FFP *fresh frozen plasma

### Renal function

We used the RIFLE criteria for evaluation of early acute renal dysfunction within 72 hours after surgery. Before matching 3.6% of men in the control group (n = 72) and 2.4% (n = 26) in the treatment group were at least at risk for AKI (difference -1.2%; p = 0.08). After matching 3.5% of men in the control and 2.5% of men in the MECC group were at least at risk for AKI (adjusted difference -1.0%; 95% CI -2.5 to 0.6; p = 0.24). The ATE was calculated as -1.2% (95% bias corrected confidence interval -2.8 to 0.04; p > 0.05 for a two-tailed test). Thus, MECC did not significantly reduce early postoperative renal dysfunction with regards to risk for AKI, kidney injury and failure.

However, significantly fewer men in the MECC group required renal replacement therapy (RRT) during the entire hospital stay (1.16% versus 2.43%; p = 0.015).

### Sensitivity analysis

Since selection bias remains the most challenging analytic problem in observational studies, we conducted a sensitivity analysis using Rosenbaum's approach. Using Wilcoxon's signed rank test, the sensitivity analysis showed that our study becomes sensitive to hidden bias at Γ = 1.5 for 30-day mortality, at Γ = 1.6 for intensive care unit stay, at Γ = 1.3 for postoperative hospital stay and for Γ = 1.6 for severe postoperative neurocognitive dysfunction. Because these values are small, we conclude that the study is very sensitive to hidden bias and therefore further analysis that controls for additional biases is warranted.

## Discussion

Propensity score methods are increasingly being used to reduce the impact of treatment-selection bias in the estimation of causal treatment effects in observational studies [16]. We used propensity score matching to estimate for the first time the treatment effect of MECC for CABG in a large sample of men. We could show that MECC significantly reduces 30-day mortality in men with an average treatment effect of -1.9%. Our results confirm previous randomized studies that showed improved survival of MECC [18,19] without differentiating between men and women. The reported ATE might seem low, but if assuming several hundred CABG in men per year, it could directly translate into many saved lives. However, even advanced statistical methods to adjust imbalance between treatment and control group in observational studies cannot compensate the current lack of at least one sufficiently powered randomized multicenter study to estimate the outcome of CABG after MECC irrespective of gender. Using our unadjusted 30-day mortality data (2.7% versus 0.8%), at least 850 male patients per group remain necessary in a randomized study to detect this difference with a power of 80% and at a significance level of 0.05. However, the mechanism why MECC might be associated with reduced mortality still is speculative and ranges from improved myocardial protection through consequent use of blood cardioplegia, reduced transfusion requirement to selection of healthier patients.

Current critics of MECC also may question whether small survival benefits justify a more complex procedure with learning curves and a more intense and challenging interplay between surgeon, anesthesiologist and perfusionist.

Analysis of other outcome variables showed that apart from postoperative neurocognitive dysfunction, intensive care stay and postoperative hospital stay largely remained uninfluenced by MECC. In addition, chest tube drainage and early postoperative kidney injury (AKI) yielded comparable results between MECC and CECC. One reason for the latter disparity may derive from different use of AKI definitions in previous studies, different protocols for extracorporeal perfusion at different institutions, but results are difficult to compare because of heterogeneous patient populations and different MECC systems.

We also could not demonstrate a lower transfusion requirement during entire hospital stay or reduced ventilation time in our sample and thus our results clearly contrast recent studies [3,20,21]. One reason originates from the many observational trials with MECC and the unavoidable selection bias in these studies, but without necessarily overestimating the magnitude of the effects of treatment. Second most of RCTs with MECC were underpowered to detect true treatment effects with regards to mortality or other "hard" endpoints because of different causes, e.g. distinctive study objectives or a low number of patients.

The clinical impact of lower creatinine kinase MB, troponin and maximal lactate release after MECC is still under debate and may rather reflect intraoperative isovolumetric perfusion than improved outcome [20,21]. The rationale for improved neurocognitive outcome for MECC patients most likely derives from improved cerebral microcirculation and decreased microembolization [22], but further research remains necessary to confirm these results.

It was assumed that different types of cardioplegia (crystalloid versus blood) were at least in part responsible for this disparity, but a recent RCT only demonstrated marginal effects [23] between both types of cardioplegia and thus its impact might be overestimated.

Although a recent meta-analysis [24] could demonstrate slightly reduced PRBC transfusion requirement and PRBC transfusion per patient, we could not confirm this findings in our large sample, in particular when the entire hospital stay is considered.

### Limitations

Findings from propensity score analyses might be potentially limited by biases related to unmeasured and hidden covariates [[16], 25]. Since our sensitivity analysis showed low Γ-values for several outcome variables, it is likely that unmeasured variables contribute to our results and require further research. Incomplete or inexact matching might also affect the results of our study. However, we could match almost 90% of our MECC patients and the overlap of propensity scores was large. This contrasts other PS studies with a matching efficiency < 60%.

## Conclusions

We observed an association between MECC use for CABG and decreased 30-day mortality by using a propensity score analysis in a large cohort of male patients. It is tempting to suggest that men should undergo elective CABG with MECC, but the findings of our study, based on a non-randomized design, are largely hypothesis generating and call for at least one randomized, multicenter clinical trial using a current risk score for precise uniform evaluation of coronary pathology and predefined criteria for perioperative care.

## Competing interests

The authors declare that they have no competing interests.

## Authors' contributions

MR: study design, data analysis and interpretation, writing manuscript; RK: study design, helping drafting the manuscript; LP: study design, helping drafting the manuscript; AK: study design, helping drafting the manuscript; MH: study design, helping drafting the manuscript; CS: study design, correction of the manuscript; CD: study design, data collection and analysis, data interpretation, helping drafting the manuscript. All authors read and approved the final manuscript.

## Pre-publication history

The pre-publication history for this paper can be accessed here:

http://www.biomedcentral.com/1471-2261/12/17/prepub

## References

[B1] Wijns W Kolh P Danchin N Di Mario C Falk V Folliguet T Garg S Huber K James S Knuuti J Lopez-Sendon J Marco J Menicanti L Ostojic M Piepoli MF Pirlet C Pomar JL Reifart N Ribichini FL Schalij MJ Sergeant P Serruys PW Silber S Sousa-Uva M Taggart D Guidelines on myocardial revascularization: the Task Force on Myocardial Revascularization of the European Society of Cardiology (ESC) and the European Association for Cardio-Thoracic Surgery (EACTS) Eur Heart J 2010 31 2501 55 20802248 10.1093/eurheartj/ehq277

[B2] Serruys PW Morice MC Kappetein AP Colombo A Holmes DR Mack MJ Ståhle E Feldman TE van den Brand M Bass EJ Van Dyck N Leadley K Dawkins KD Mohr FW Percutaneous coronary intervention versus coronary-artery bypass grafting for severe coronary artery disease N Engl J Med 2009 360 961 972 10.1056/NEJMoa0804626 19228612

[B3] Wiesenack C Liebold A Philipp A Ritzka M Koppenberg J Birnbaum DE Keyl C Four years' experience with a miniaturized extracorporeal circulation system and its influence on clinical outcome Artif Organs 2004 28 1082 1088 10.1111/j.1525-1594.2004.00030.x 15554936

[B4] Fromes Y Gaillard D Ponzio O Chauffert M Gerhardt MF Deleuze P Bical OM Reduction of the inflammatory response following coronary bypass grafting with total minimal extracorporeal circulation Eur J Cardiothorac Surg 2002 22 527 533 10.1016/S1010-7940(02)00372-X 12297167

[B5] Schottler J Lutter G Boning A Soltau D Bein B Caliebe D Haake N Schoeneich F Cremer J Is there really a clinical benefit of using minimized extracorporeal circulation for coronary artery bypass grafting? Thorac Cardiovasc Surg 2008 56 65 70 10.1055/s-2007-989336 18278679

[B6] Biancari F Rimpilainen R Meta-analysis of randomised trials comparing the effectiveness of miniaturised versus conventional cardiopulmonary bypass in adult cardiac surgery Heart 2009 95 964 969 10.1136/hrt.2008.158709 19342377

[B7] Puehler T Haneya A Philipp A Camboni D Hirt S Zink W Lehle K Rupprecht L Kobuch R Diez C Schmid C Minimized extracorporeal circulation in coronary artery bypass surgery is equivalent to standard extracorporeal circulation in patients with reduced left ventricular function Thorac Cardiovasc Surg 2010 58 204 209 10.1055/s-0029-1241028 20514574

[B8] Blankstein R Ward RP Arnsdorf M Jones B Lou YB Pine M Female gender is an independent predictor of operative mortality after coronary artery bypass graft surgery: contemporary analysis of 31 Midwestern hospitals Circulation 2005 112 I323 I327 16159840 10.1161/CIRCULATIONAHA.104.525139

[B9] Blasberg JD Schwartz GS Balaram SK The role of gender in coronary surgery Eur J Cardiothorac Surg 2011 40 715 721 21349733 10.1016/j.ejcts.2011.01.003

[B10] Koch CG Khandwala F Nussmeier N Blackstone EH Gender and outcomes after coronary artery bypass grafting: a propensity-matched comparison J Thorac Cardiovasc Surg 2003 126 2032 2043 10.1016/S0022-5223(03)00950-4 14688723

[B11] Roques F Michel P Goldstone AR Nashef SA The logistic EuroSCORE Eur Heart J 2003 24 881 882 10.1016/S0195-668X(02)00801-1 12727160

[B12] Levey AS Greene T Schluchter MD Cleary PA Teschan PE Lorenz RA Molitch ME Mitch WE Siebert C Hall PM Glomerular filtration rate measurements in clinical trials. Modification of Diet in Renal Disease Study Group and the Diabetes Control and Complications Trial Research Group J Am Soc Nephrol 1993 4 1159 1171 8305642 10.1681/asn.v451159 PMC2866096

[B13] Bellomo R Ronco C Kellum JA Mehta RL Palevsky P Acute renal failure-definition, outcome measures, animal models, fluid therapy and information technology needs: the Second International Consensus Conference of the Acute Dialysis Quality Initiative (ADQI) Group Crit Care 2004 8 R204 R212 10.1186/cc2872 15312219 PMC522841

[B14] Puehler T Haneya A Philipp A Zausig YA Kobuch R Diez C Birnbaum DE Schmid C Minimized extracorporeal circulation system in coronary artery bypass surgery: a 10-year single-center experience with 2243 patients Eur J Cardiothorac Surg 2011 39 459 464 10.1016/j.ejcts.2010.08.006 20851618

[B15] Austin PC Propensity-score matching in the cardiovascular surgery literature from 2004 to 2006: a systematic review and suggestions for improvement J Thorac Cardiovasc Surg 2007 134 1128 1135 10.1016/j.jtcvs.2007.07.021 17976439

[B16] Ohata T Mitsuno M Yamamura M Tanaka H Kobayashi Y Ryomoto M Yoshioka Y Tsujiya N Miyamoto Y Beneficial effects of mini-cardiopulmonary bypass on hemostasis in coronary artery bypass grafting: analysis of inflammatory response and hemodilution ASAIO J 2008 54 207 209 10.1097/MAT.0b013e3181648dbc 18356657

[B17] Remadi JP Rakotoarivelo Z Marticho P Benamar A Prospective randomized study comparing coronary artery bypass grafting with the new mini-extracorporeal circulation Jostra System or with a standard cardiopulmonary bypass Am Heart J 2006 151 198 16368318 10.1016/j.ahj.2005.03.067

[B18] Huybregts RA Morariu AM Rakhorst G Spiegelenberg SR Romijn HW de Vroege R van Oeveren W Attenuated renal and intestinal injury after use of a mini-cardiopulmonary bypass system Ann Thorac Surg 2007 83 1760 1766 10.1016/j.athoracsur.2007.02.016 17462395

[B19] Beghi C Nicolini F Agostinelli A Borrello B Budillon AM Bacciottini F Friggeri M Costa A Belli L Battistelli L Gherli T Mini-cardiopulmonary bypass system: results of a prospective randomized study Ann Thorac Surg 2006 81 1396 1400 10.1016/j.athoracsur.2005.10.015 16564279

[B20] Liebold A Khosravi A Westphal B Skrabal C Choi YH Stamm C Kaminski A Alms A Birken T Zurakowski D Steinhoff G Effect of closed minimized cardiopulmonary bypass on cerebral tissue oxygenation and microembolization J Thorac Cardiovasc Surg 2006 131 268 276 10.1016/j.jtcvs.2005.09.023 16434253

[B21] Ovrum E Tangen G Tollofsrud S Oystese R Ringdal MA Istad R Cold blood cardioplegia versus cold crystalloid cardioplegia: a prospective randomized study of 1440 patients undergoing coronary artery bypass grafting J Thorac Cardiovasc Surg 2004 128 860 865 10.1016/j.jtcvs.2004.03.032 15573070

[B22] Benedetto U Angeloni E Refice S Capuano F Goracci M Roscitano A Is minimized extracorporeal circulation effective to reduce the need for red blood cell transfusion in coronary artery bypass grafting? Meta-analysis of randomized controlled trials J Thorac Cardiovasc Surg 2009 138 1450 1453 10.1016/j.jtcvs.2009.03.042 19660383

[B23] D'Agostino RB Jr Propensity scores in cardiovascular research Circulation 2007 115 2340 2343 10.1161/CIRCULATIONAHA.105.594952 17470708

